# Chemical Exchange Saturation Transfer for Lactate-Weighted Imaging at 3 T MRI: Comprehensive In Silico, In Vitro, In Situ, and In Vivo Evaluations

**DOI:** 10.3390/tomography8030106

**Published:** 2022-05-07

**Authors:** Karl Ludger Radke, Daniel B. Abrar, Miriam Frenken, Lena Marie Wilms, Benedikt Kamp, Matthias Boschheidgen, Patrick Liebig, Alexandra Ljimani, Timm Joachim Filler, Gerald Antoch, Sven Nebelung, Hans-Jörg Wittsack, Anja Müller-Lutz

**Affiliations:** 1 Department of Diagnostic and Interventional Radiology, Medical Faculty, University Dusseldorf, D-40225 Dusseldorf, Germany; ludger.radke@med.uni-duesseldorf.de (K.L.R.); miriam.frenken@med.uni-duesseldorf.de (M.F.); lena.wilms@med.uni-duesseldorf.de (L.M.W.); benedikt.kamp@med.uni-duesseldorf.de (B.K.); matthias.boschheidgen@med.uni-duesseldorf.de (M.B.); alexandra.ljimani@med.uni-duesseldorf.de (A.L.); antoch@med.uni-duesseldorf.de (G.A.); snebelung@ukaachen.de (S.N.); hans-joerg.wittsack@med.uni-duesseldorf.de (H.-J.W.); anja.lutz@med.uni-duesseldorf.de (A.M.-L.); 2 Siemens Healthcare GmbH, D-91052 Erlangen, Germany; patrick.liebig@siemens-healthineers.com; 3 Institute of Anatomy I, Heinrich-Heine-University, D-40225 Dusseldorf, Germany; timm.filler@uni-duesseldorf.de; 4 Department of Diagnostic and Interventional Radiology, University Hospital Aachen, D-52074 Aachen, Germany

**Keywords:** magnetic resonance imaging, molecular imaging, simulation, CEST, LATEST, lactate, in silico, in vitro, in situ, in vivo

## Abstract

Based on in silico, in vitro, in situ, and in vivo evaluations, this study aims to establish and optimize the chemical exchange saturation transfer (CEST) imaging of lactate (Lactate-CEST—LATEST). To this end, we optimized LATEST sequences using Bloch–McConnell simulations for optimal detection of lactate with a clinical 3 T MRI scanner. The optimized sequences were used to image variable lactate concentrations in vitro (using phantom measurements), in situ (using nine human cadaveric lower leg specimens), and in vivo (using four healthy volunteers after exertional exercise) that were then statistically analyzed using the non-parametric Friedman test and Kendall Tau-b rank correlation. Within the simulated Bloch–McConnell equations framework, the magnetization transfer ratio asymmetry (MTR_asym_) value was quantified as 0.4% in the lactate-specific range of 0.5–1 ppm, both in vitro and in situ, and served as the imaging surrogate of the lactate level. In situ, significant differences (*p* < 0.001) and strong correlations (τ = 0.67) were observed between the MTR_asym_ values and standardized intra-muscular lactate concentrations. In vivo, a temporary increase in the MTR_asym_ values was detected after exertional exercise. In this bench-to-bedside comprehensive feasibility study, different lactate concentrations were detected using an optimized LATEST imaging protocol in vitro, in situ, and in vivo at 3 T, which prospectively paves the way towards non-invasive quantification and monitoring of lactate levels across a broad spectrum of diseases.

## 1. Introduction

Non-invasive imaging of lactate is essential for the early diagnosis of diseases associated with altered physiological energy metabolism [1]. Such diseases include cancer [2,3], heart failure [4,5], diabetes [6,7], sepsis [8,9], myositis [10], and neurologic disorders such as ischemic stroke [11,12,13,14]. In athletes, lactate measurements after exertion provide information about the individual athlete’s metabolism that may help guide training, especially in elite sports [15].

Cancer cells frequently exhibit anaerobic lactic glycose metabolism due to enzymatic upregulation [2]. Thus, significantly increased lactate levels occur in the vicinity of cancer [16]. In previous studies, elevated lactate levels have been correlated with an increased risk of metastases [3]. However, lactate is also an important biomarker in pediatric and neurologic pathologies [1,17]. Therefore, non-invasive measurement of lactate is essential for investigating metabolic alterations in a variety of pathologies. Lactate was thus established as an essential biomarker in routine clinical practice for the diagnosis of sepsis and has been included in its definition since 2016 [18]. Furthermore, cerebrospinal fluid lactate levels proved to be an independent predictor of stroke outcome [13,19]. Previous studies have shown that myositis is associated with changes in serum concentrations of various enzymes and metabolites such as lactate [10,20]. Currently, the diagnosis of myositis relies on histopathologic evaluation via muscle biopsy that necessarily requires the identification of the muscle portion that is most severely affected. Morphologic magnetic resonance imaging (MRI) techniques are used to identify and localize muscle edema associated with inflammation [20]. Lactate imaging could complement morphologic MRI to avoid false-negative biopsies.

Biochemically sensitive imaging of lactate would thus cover a broad medical spectrum and aid in the early diagnosis of various diseases. Currently, there are two main methods of lactate measurement by MR. One is conventional MR spectroscopy (MRS; both ^1^H and ^13^C), used to detect abnormal lactate accumulation as a significant lactate peak [21,22,23]. The other method involves imaging with infused 13-labeled pyruvate [24,25]. Imaging with pyruvate provides a more than 10,000-fold signal enhancement as compared to MRS but is invasive and requires specialized equipment. Chemical exchange saturation transfer (CEST) imaging is increasingly used as an extension of MRS. CEST combines the advantages of MRS and MRI [26]. During CEST imaging, exogenous or endogenous molecules with exchangeable protons, such as hydroxyl protons (-OH), amine protons (-NH_2_), and amide protons (-NH), are selectively saturated, and the exchanges with water protons are detected [27]. Furthermore, due to a large number of multiple proton exchanges during the saturation time, CEST is characterized by higher sensitivity than MRS [28].

The chemical OH-proton shift of lactate is in the range of 0.4–0.8 ppm [16]. Therefore, optimized lactate CEST sequences that account for various overlying saturation effects, such as direct water saturation, are essential for direct detection of lactate. Nevertheless, DeBrosse et al. used lactate CEST imaging (LATEST) to detect lactate increases in lymphoma flank cancer in animal models, and after athletic exercise in the human calf at 7 T [16]. However, in recent years, CEST imaging has also been successfully applied at 3 T with OH-protons of other metabolites such as glycosaminoglycans to detect degenerative joint diseases and age-related degeneration of articular cartilage and intervertebral discs [29,30,31]. Furthermore, CEST imaging has recently been used to detect exercise-induced physiological changes in the lower back of elite rowing athletes [32]. Thus, CEST imaging, along with T1rho (T_1ρ_) imaging, diffusion tensor imaging (DTI), and sodium imaging (^23^Na), has been established as a biochemically sensitive method to detect early physiological changes using MRI [33,34,35].

High-field MRI systems working at field strengths of 7 T or more are largely unavailable for the clinical routine. To our knowledge, there are no studies that have investigated the feasibility of LATEST imaging at a clinical field strength of 3 T. Therefore, this study aimed to fill this gap and to study the potential of non-invasively differentiating lactate concentrations using LATEST imaging and a range of variable configurations that balance experimental standardization and clinical applicability from bench to bedside. To this end, we hypothesized that (1) based on an imaging protocol optimized in silico, different lactate concentrations may be detected with LATEST imaging in vitro and in situ; (2) for LATEST imaging, the optimal pre-saturation modulus and detected asymmetric magnetization transfer ratios (MTR_asym_) values are temperature dependent; and (3) in vivo, an increase in muscle lactate concentration after “all-out” athletic exercise can be detected with a CEST imaging protocol optimized for in vivo measurements.

## 2. Materials and Methods

### 2.1. Study Design

This study was designed as an exploratory feasibility analysis involving successive in vitro, in situ, and in vivo CEST-MRI evaluations of lactate levels (after numerical sequence optimization). It was organized in three phases: (1) numerical simulations to optimize pre-saturation modules for LATEST imaging based on the Bloch–McConnell equations, (2) evaluation of the optimized pre-saturation modules for LATEST imaging in vitro and in situ under systematic variation of lactate concentrations, and (3) demonstration of principal applicability of LATEST imaging in vivo before and after athletic exercise.

Written informed consent was obtained from all body donors as well as from the healthy volunteers. The study was approved by the local ethics committee (Ethical Committee of the Medical Faculty of Heinrich-Heine-University, Düsseldorf, Germany, Study numbers: in situ 2021-1487 and in vivo 2021-1528).

### 2.2. MRI

All MRI measurements were performed on a clinical 3 T MRI scanner (MAGNETOM Prisma, Siemens Healthineers, Erlangen, Germany) with a dedicated 15-channel knee coil (Tx/Rx Knee 15 Flare Coil, Siemens Healthineers).

The MRI protocol included standard morphologic sequences, i.e., coronal T2-weighted (T2w) turbo spin-echo (TSE) and sagittal T1-weighted (T1w) TSE sequences. Further MR mapping acquisitions were acquired to map relaxation times: For T1 mapping, an inversion recovery TSE sequence was used with seven inversion times (TIs: 25–3000 ms), while for T2 mapping, a spin-echo (SE) sequence was acquired with 16 different echo times (TEs: 9.7–164.9 ms) as well as a LATEST CEST sequence (WIP 816 A, Siemens Healthineers). The pre-saturation module of our prototype LATEST CEST sequence used Hanning-windowed Gaussian high-frequency pulses. A total of 62 pre-saturated images were acquired at different saturation frequencies symmetric around the water resonance, with a reference image at 300 ppm. The maximum frequency deviation was Δω = 2.5 ppm. The high-frequency field strengths (B_1_ = 0.6 µT or 0.5 µT), pulse duration (t_p_ = 50 ms), inter-pulse delay (t_d_ = 50 ms), and number of pre-saturation pulses (n_p_ = 8 or 15) were set as derived from the optimized Bloch–McConnell results (see below). For B_0_ inhomogeneity correction, a Water Saturation Shift Referencing (WASSR) acquisition was performed with t_p_ = 25 ms, t_d_ = 25 ms, n_p_ = 1, B_1_ = 0.2 µT, 62 dynamics, and Δω = 1 ppm; otherwise, the same sequence parameters as for the CEST sequence were used. Further image parameters are indicated in Table 1.

### 2.3. In Silico Study: Numerical Sequence Optimization

The efficient selective saturation of exchanging protons is required to observe CEST effects. Bloch–McConnell equations can describe the exchange processes of different spin systems [36]. The first phase of our study was aimed to optimize sequence settings (i.e., B_1_, t_p_, and n_p_), respecting scanner-specific constraints as well as specific absorption rate (SAR) limits. For simulations, the MATLAB (MatlabR2020b, Natick, MA, USA) script published by Zaiss et al. was used, which can be downloaded at https://github.com/cest-sources/BM_sim_fit/ (accessed on 16 March 2021) and has been validated in numerous studies [31,37,38,39].

Simulations were performed considering the water and lactate pool. The water pool parameters T_1_ and T_2_ were determined prior to this study in pilot measurements using the lower legs (gastrocnemius muscle) of a human cadaveric specimen (right side, 78 years of age) and a healthy volunteer (right side, 26 years of age) as T_1,in-situ_ = 856 ± 34 ms, T_2,in-situ_ = 69 ± 4 ms and T_1,in-vivo_ = 1412 ± 49 ms, T_2,in-vivo_ = 50 ± 8 ms. For lactate OH-protons, we used temperature-dependent exchange rates (k_sw_) of ~350 Hz (at 20 °C) and ~550 Hz (at 37 °C), as well as temperature-dependent chemical shifts between 0.4 ppm (at 4 °C) and 0.8 (at 37 °C) ppm, as shown in previous studies [16]. In addition, a water concentration of 88 M [31], and a lactate concentration of 15 mM (expected lactate concentration in cancer tissue [40] and after athletic exercise [16]) were assumed. The other simulation parameters, as well as the simulation limits, are shown in Table 2.

Subsequently, optimal sequence settings were calculated using a constant duty cycle of 0.5 as a constrained nonlinear multivariable minimization problem. For minimization of the parameter crit (Equation (1)), the range of normalized direct water saturation ($\bar{\Delta \mathrm{Spillover}})$, normalized MTR_asym_ effect (${\bar{\mathrm{MTR}}}_{\mathrm{asym}})$, and maximum MTR_asym_ position (${\bar{\Delta \omega }}_{\mathrm{Lactate}-\mathrm{OH}\;\mathrm{Peak}})$ were used:

(1) $$\mathrm{crit}\left(B_{1},\mathrm{tp},n_{p}\right)=\frac{\bar{\Delta \mathrm{Spillover}}}{{\bar{\mathrm{MTR}}}_{\mathrm{asym}}+{\bar{\Delta \omega }}_{\mathrm{Lactate}-\mathrm{OH}\;\mathrm{Peak}}}$$

### 2.4. In Vitro Study

**Preparation of phantoms**: For the concentration-dependent phantom study, calcium L-(+)-lactate hydrate (Carl ROTH, Karlsruhe, Germany) was dissolved in phosphate-buffered saline (ROTI^®^Cell PBS, Carl ROTH) at pH 7.3 (pH value of a muscle at rest [41]), and phantoms were prepared with different concentrations of lactate, i.e., 0, 5, 10, 20, and 40 mM lactate. For the pH-dependent phantom study, 40 mM lactate samples were prepared in PBS solutions of pH values with 7.6, 7.3, 7.0, and 6.7.

**In vitro Phantom Measurements**: A custom-made MR-compatible phantom with eight holding fixtures for test tubes was used (Figure S1). In addition, the phantom had a 450 mL water reservoir for temperature adjustment and was measured at room temperature as well as at 37 °C. To this end, we centered the phantom in the knee coil and MRI scanner. CEST imaging was performed with sequence settings optimized in our in silico studies for both in situ (room temperature) and in vivo (37 °C) parameters. B_0_ inhomogeneity correction was performed using the WASSR sequence. For monitoring of the adjusted temperature (37 °C), a standard thermometer (DIGItemp, Servopray GmbH, Wesel, Germany) was used.

### 2.5. In Situ Study

Human cadaveric lower leg specimens: The local Institute of Anatomy I (Heinrich Heine University, Düsseldorf, Germany) provided nine fresh-frozen human lower leg specimens for the in situ measurements. The mean age of the body donors at the time of death was 81 ± 9 years (range 71–96 years), of which eight were female and one was male, while five were left and four were right specimens. Before each MRI examination, specimens were thawed and warmed to room temperature for at least 24 h.

**Figure 1:**
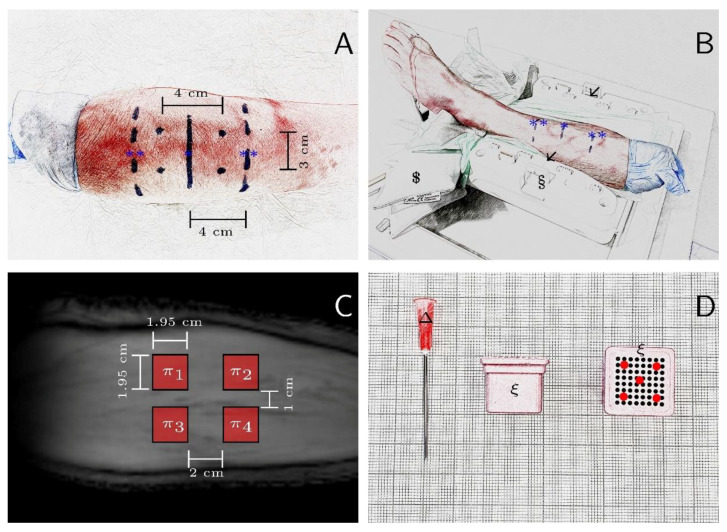
Experimental setup and sample preparation to investigate CEST metabolite sensitivity in situ using human cadaveric lower leg specimens. (**A**) For preparation, the specimens were marked to standardize lactate measurement sites. The en face view of the human calf where left is proximal, and right is distal. Marker lines indicate the widest part of the gastrocnemius muscle (*, solid line), and two parallel marker lines at a distance of 4 cm indicate accessory lines (**, dashed lines). (**B**) Specimens were placed supine and feet first in a clinical knee coil (§) (which is open for better illustration) and fixed using mechanical positioning aids such as sandbags ($). The marker lines were used to standardize specimen placement in the coil along the reference lines (black arrows). (**C**) Overlay of the four standardized regions of interest (π_1–4_) on the CEST reference image (300 ppm). (**D**) Grid needle blocks (ξ) viewed from the side and en face as well as the 18G needle (Δ) on graph paper. To standardize intramuscular injections of lactate in terms of position (red dots) and depth (needle was fully inserted into the needle block), needle blocks were used.

In practical terms, D.B.A. (radiologist with six years of experience in musculoskeletal imaging) prepared the specimens by circumferentially marking the widest part of gastrocnemius muscle and two parallel lines at an equidistance of 4 cm (Figure 1A,B). A 3 cm × 4 cm rectangle was centered around the central marker line on the calf and marked along its corners (Figure 1A—black dots). The marked points and lines served as orientation for standardized positioning of the four regions of interest (Figure 1C) and of the specimens in the knee coil (Figure 1B). Then, 18G grid needle blocks (Noras MRI product GmbH, Höchberg, Germany) were used to standardize intramuscular injections of lactate in terms of position and depth (Figure 1D). Each region of interest (π_1–4_ in Figure 1C) was injected with 1 mL altogether (i.e., 0.2 mL at each of the five positions as indicated by the red dots in Figure 1D) using a standard 18G needle (BDTM Blunt Fill Needle, Becton Deickinsson, Franklin Lakes, NJ, US), which was fully inserted into the needle block. Calcium L-(+)-lactate hydrate was dissolved in Ampuwa, analogous to the in vitro study, and concentrations of 0, 20, 100, and 200 mM were prepared. Ampuwa is highly pure deionized water; due to the resulting high osmotic gradient, injected concentrations were homogeneously distributed in the study volume (4 cm^3^). Consequently, concentrations of 0, 5, 20, and 40 mM were present in the gastrocnemius muscle.

In situ MR imaging: All specimens were placed in the knee coil in the feet first and supine position with standardization realized by using the marker lines (Figure 1B). In addition, mechanical positioning fixtures such as sandbags were used. Based on the numerical simulation results, B_1_ = 0.6 µT, t_p_ = 50 ms, and n_p_ = 8 were used for the CEST pre-saturation modulus. Human cadaveric specimens were examined before and after lactate injections. A WASSR sequence as described above was used for B_0_ correction.

### 2.6. In Vivo Study

The in vivo study was performed on the right calves of four healthy volunteers, three male and one female (mean age 26.0 ± 2.2 years; range 24–29 years). D.B.A. marked the lower legs as described above, and the volunteers were positioned as the specimens. Baseline sequences (i.e., morphologic sequences, T_1_ and T_2_ mapping) were obtained before athletic exercise as previously described. According to the numerical simulations, CEST parameters were selected as B_1_ = 0.5 µT, t_p_ = 50 ms, and n_p_ = 15. Repetitious vigorous plantarflexion and dorsiflexion exercises were performed until exhaustion (~3 min) [16], but before any muscle cramps or spasms. Immediately after exhaustion and for a period of an additional 22 min, CEST measurements were continuously acquired, resulting in a total of five measurements altogether.

### 2.7. MR Image Analysis

A Python (v3.8.4, Python Software Foundation, Wilmington, DE, USA) script written in house and based on cross-correlation analysis in Fourier space [42] was used to correct the position of the lower leg in the in situ and in vivo measurements between time points, i.e., pre- and post-injection (in situ) and pre- and post-exercise (in vivo). Subsequently, D.B.A. labelled the four regions of interest of the in situ measurements (π_1–4_ in Figure 1C) and a 6.2 cm × 6.2 cm square region centrally in gastrocnemius muscle for the in vivo measurement.

Further analysis of ultrastructural and compositional tissue properties was carried out using MATLAB scripts (implemented and validated in house) as well as the labelled ROI [30,31]. T1 and T2 relaxation times were calculated pixel-wise. Offset maps were calculated based on the WASSR measurements. The CEST data were normalized to the signal from the first acquired image at a frequency offset of 300 ppm. The offset maps were used to correct Z-spectra. The CEST asymmetry was evaluated using MTR_asym_ [43]. Frequency ranges (0–0.5, 0.5–1, 1–1.5, and 1.5–2 ppm) were analyzed. The MTR_asym_ values for the respective ranges given below correspond to the mean value.

### 2.8. Statistical Analyses

Statistical analyses were performed by K.L.R. in R (v4.0.3, R Foundation for Statistical Computing, Vienna, Austria) [44] using the packages “pgirmess” [45], “pysch” [46], and “ggpubr” [47]. For the in vitro and in vivo measurements, descriptive statistics were used, while for the in situ measurements, non-parametric tests were used because of the small sample size. In the following, δ_0_ refers to the baseline values (pre-injection), while δ_1_ refers to the follow-up values (post-injection). Mean T_1_ and T_2_ relaxation times δ_0_ were compared as a function of concentration using the Friedman test. To reduce the number of statistical tests as well as the individual influence of initially different lactate levels in the muscle, the absolute changes (ΔX = X[δ_1_] − X[δ_0_]) were calculated. Then, absolute changes of MTR_asym_ values were compared between the different lactate concentrations using Friedman tests followed by Wilcoxon signed-rank post hoc tests (alpha correction according to Bonferroni–Holm method [48]), if necessary. To study possible statistical associations between relative changes and injected lactate concentrations, the Kendall Tau-b rank correlation coefficient was determined. The effect size tau was classified as small (0.1–0.3), medium (0.3–0.5), and strong (>0.5), according to Cohen et al. [49].

Due to the exploratory design of this study and the small sample size, the significance level was set to *p* ≤ 0.01. Alpha error correction was performed according to the Bonferroni–Holm method [48], if necessary. This “low” significance level was chosen to prevent inflation of alpha error while maintaining statistical power. Unless other specified, data are given as means ± standard deviations.

## 3. Results

### 3.1. In Silico Study: Numerical Sequence Optimization

**Figure 2:**
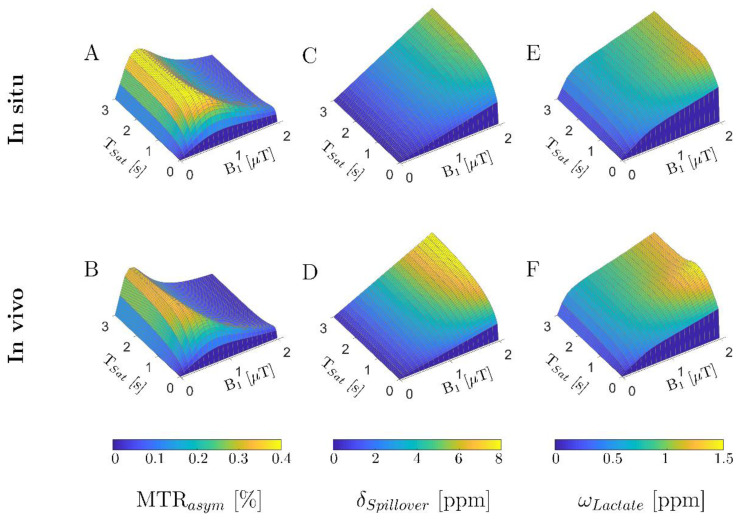
Three-dimensional plots of saturation times (T_sat_) and radiofrequency field strengths (B_1_) using simulation frameworks for in situ (T_1_ = 856 ms, T_2_ = 69 ms, and k_sw_ = 350 Hz) and in vivo (T_1_ = 1412 ms, T_2_ = 50 ms, and k_sw_ = 550 Hz) pulse train CEST simulations. (**A**,**B**) Simulated maximum CEST effect as indicated by MTR_asym_; (**C**,**D**) simulated width of direct water saturation as indicated by δ_Spillover_; (**E**,**F**) location of maximum simulated lactate CEST effect as indicated by ω_Lactate_.

For the parameters of the in situ and in vivo simulations, increases of the CEST effect with saturation time (T_Sat_) were observed, with a substantial change up to a duration of 0.4 s (in situ) and 0.7 s (in vivo) before stabilizing at 0.4% MTR_asym_ (in situ) and 0.25% MTR_asym_ (in vivo), respectively (Figure 2A,B). In comparison, with a variation of B_1_, initial increases of MTR_asym_ values up to B_1_ = 0.6 µT were observed but decreased with a further increase of B_1_. In addition, a monotonically increasing width of direct water saturation with B_1_ and T_Sat_ (Figure 2C,D) and an associated shift of the maximum CEST effect toward 1 ppm were observed (Figure 2E,F).

Due to SAR limits, T_Sat_ must be divided into several shorter pulses. Considering the above-mentioned effect as well as determined relaxation times, SAR limits, and temperature-dependent exchange rates, optimal sequence settings were found for in situ with B_1_ = 0.6 µT, t_p_ = 50 ms, and n_p_ = 8 as well as for in vivo with B_1_ = 0.5 µT, t_p_ = 50 ms, and n_p_ = 15.

### 3.2. In Vitro Study

**Figure 3:**
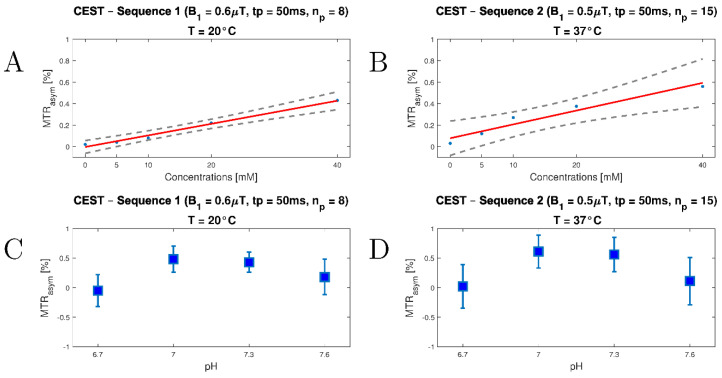
Plots to visualize MTR_asym_ values (MTR_asym_) as a function of lactate concentration and pH value for two CEST sequences (i.e., CEST-Sequence 1 for future in situ use with B1 = 0.6 µT, tp = 50 ms, and np = 8; CEST-Sequence 2 for future in vivo use with B1 = 0.5 µT, tp = 50 ms, and np = 15) at (**A**,**C**) room temperature and (**B**,**D**) 37 °C. In addition to MTR_asym_ (blue dots), linear regression (red continuous line) and 95% confidence intervals (grey dashed lines) are plotted for concentration dependence. For the pH dependence, the values are given as error plots with mean and standard deviation. Z-spectra were corrected using WASSR correction, and temperature was stabilized at 37 °C using a 450 mL water bath.

Increasing MTR_asym_ values as a function of lactate concentration were measured for both CEST sequences (Figure 3A,B and Figure S2A,B). The maximum MTR_asym_ value was 0.43% at room temperature and 0.56% at 37 °C, measured at 40 mM and pH 7.3. We also observed a temperature-independent lower detection limit of about 5–10 mM. For both in vitro experiments, the highest MTR_asym_ values were observed at pH = 7.0 (Figure 3C,D). We observed an increase in MTR_asym_ value from 0.56% to 0.61% at a temperature of 37 °C and a decrease in pH from 7.3 to 7.0. However, when the pH was lowered further (pH = 6.7), the CEST effect decreased substantially, as it did when the pH was increased to 7.6. We monitored the temperature before (T = 37.1 °C) and after the measurement (T = 36.7 °C).

### 3.3. In Situ Study

**T_1_ and T_2_ relaxation times:** At baseline (δ_0_), mean T_1_ (*p* = 0.435) and T_2_ relaxation times (*p* = 0.204) showed no significant differences for the four regions of interest (π_1–4_). Furthermore, lactate injection had no effect on T_1_ (*p* = 0.338) or T_2_ relaxation times (*p* = 0.046). Mean T_1_ relaxation times were 824 ± 153 ms (δ_0_) and 832 ± 151 ms (δ_1_), respectively, and mean T_2_ relaxation times were 70 ± 9.0 ms (δ_0_) and 72 ± 9.4 ms (δ_1_), respectively. 

**Figure 4:**
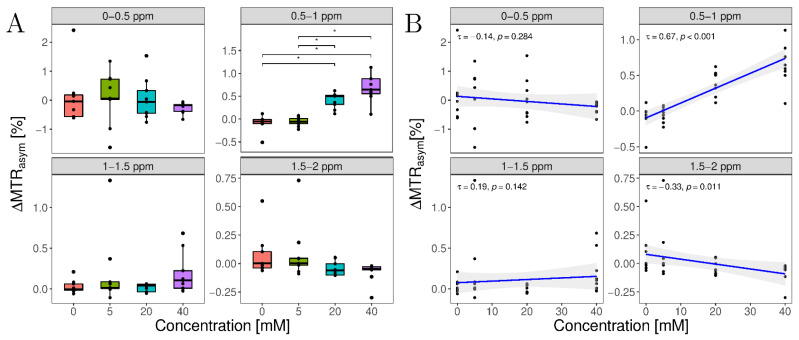
Visualization of absolute changes of the asymmetric magnetization transfer ratios (ΔMTR_asym_) as a function of injected lactate concentrations as (**A**) box plots and (**B**) scatter plots with correlations. (**A**) Boxplots of ΔMTR_asym_ values as a function of systematically varied assessment ranges. Thick lines indicate medians, while box outlines indicate the upper (75th percentile, Q_3_) and lower quartiles (25th percentile, Q_1_), and whiskers with a maximum width of 1.5 Interquartile range (IQR = Q_3_–Q_1_). All other observed points are plotted as outliers. Significant differences are marked by asterisks (*) (**B**) Correlations between ΔMTR_asym_ and injected lactate concentrations as function of systematically varied assessment ranges.

**MTR_asym_ analysis:** Significant differences (*p* < 0.001) in ΔMTR_asym_ values were found for the lactate-specific range of 0.5–1 ppm, while other ranges did not display significant differences (Table 3). Post hoc analyses showed that injected lactate concentrations of 0 vs. 20 mM, 0 vs. 40 mM, 5 vs. 20 mM, and 5 vs. 40 mM were significantly different (*p* < 0.01) (as indicated by the asterisks in Figure 4A). Moreover, ΔMTR_asym_ values showed a strong and significant (τ = 0.67, *p* < 0.001) correlation with the injected lactate concentration (Figure 4B). In contrast, no significant differences (Table 3 and Figure 4A) and no significant correlations were observed for the other ranges 0–0.5 ppm (*p* = 0.28), 1–1.5 ppm (*p* = 0.14), and 1.5–2 ppm (*p* = 0.011) (Figure 4B). Consequently, this was also reflected in the MTR_asym_ curves as a significant increase in the MTR_asym_ peak in the range of 0.4–1.2 ppm compared with the pre- and post-lactate injection (Figure S2C,D).

### 3.4. In Vivo Study

**T_1_ and T_2_ relaxation times:** Mean T_1_ and T_2_ relaxation times of the healthy volunteers at baseline were T_1_ = 1915 ± 5 ms, and T_2_ = 41 ± 3 ms, within the value ranges assumed for the corresponding Bloch–McConnell simulations.

**Figure 5:**
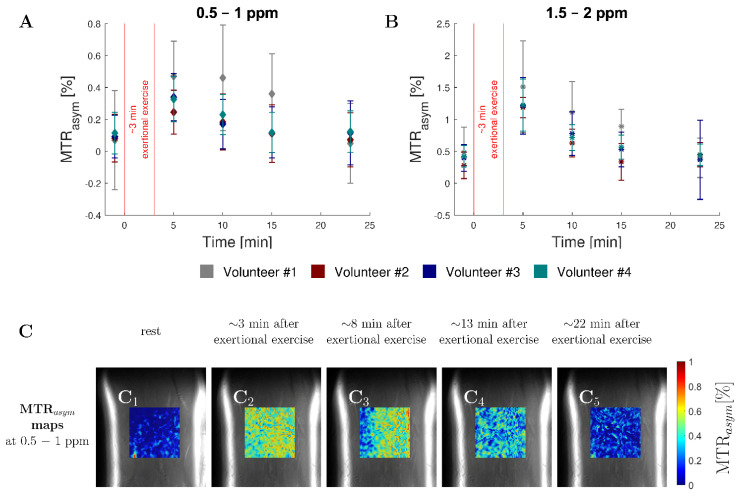
Longitudinal changes of MTR_asym_ values (given as mean and standard deviation) of the four human calves before and immediately after exertional exercise. (**A**) Immediately after exercise, we observed in all volunteers (different colors) a substantial increase in MTR_asym_ values in the lactate-specific range of 0.5–1 ppm. During the following post-exercise period, this increase gradually equilibrated to reach the resting state. (**B**) Similarly, in the creatine-specific range of 1.5–2 ppm, we observed a corresponding increase and decrease in MTR_asym_ values following exertional exercise. (**C**) Morphologic images of a representative human calf overlaid with the MTR_asym_ maps in the range 0.5–1 ppm with successive time points indicated by C_1_ to C_5_.

**MTR_asym_ Analysis:** The healthy human calves showed mean MTR_asym_ values of 0.09 ± 0.02% in the lactate-specific range of 0.5–1 ppm at rest. Immediately after exertional exercise (of ~3 min), the asymmetry increased to 0.34 ± 0.09% and constantly decreased to the resting value of 0.09 ± 0.04% during the following 20 min (Figure 5A,C). In addition, corresponding changes in the frequency range of creatin (1.5–2 ppm) were recorded. In this range, the asymmetry changed from 0.23 ± 0.09% at rest to 0.62 ± 0.05% immediately after exercise. As in the lactate-specific range, a continuous decrease was observed in the following 22 min to a resting value of 0.28 ± 0.10% (Figure 5B), although in this range, a much faster decrease to resting value was observed. In contrast, the other evaluated ranges (of 0–0.5 ppm and 1–1.5 ppm, respectively) showed no such changes (Figure S3).

## 4. Discussion

The most important finding of our study is that LATEST imaging is feasible using a standard clinical 3 T MRI scanner after extensive numerical sequence optimization. In addition, we demonstrated a strong correlation of the MTR_asym_ values as a function of lactate concentration in vitro (in a phantom) and in situ (in human cadaveric specimens). Furthermore, we observed a temporary change of the MTR_asym_ values as surrogate for lactate in vivo after exertional exercise.

Compositional MRI goes beyond the mere morphologic depiction of tissue. It enables the compositional assessment of tissue and the early detection of biochemical alterations that precede morphologic changes [50]. Lactate is an end product of anaerobic glycolysis and, therefore, an important marker of various diseases associated with altered biochemical energy supply [1]. Blood lactate levels are the most commonly analyzed parameter; however, they do not provide information on where exactly lactate is produced. To determine the origins of altered metabolism and focal changes of lactate concentration is of clinical interest. Despite this clinical need to determine locally altered lactate levels, LATEST imaging research is generally limited by numerous technical difficulties such as magnetic field homogeneity, low signal-to-noise ratios (SNRs), and the low chemical shift of OH-protons relative to water. Nevertheless, LATEST imaging has been successfully evaluated at 7 T and 9.4 T recently [16]. 

LATEST imaging depends on several parameters such as the number of exciting saturation pulses, the pulse duration, and the high-frequency field strength. In this context, exchange processes crucial for efficient and selective saturation can be described by the Bloch–McConnell equations [36]. The CEST pre-saturation modulus was determined as a function of tissue-specific relaxation times, exchange rate, and temperature as a numerical optimization problem to find the optimal setting of these parameters. As in previous studies, our simulations showed an increase of MTR_asym_ with saturation time [31,51]. Due to SAR constraints, the saturation pulse duration was distributed over multiple pulses with a maximum of 100 ms, analogous to previous studies [51,52]. Here, our simulation results show that it is more reasonable to minimize the pulse duration further (t_p_ = 50 ms) and split it into multiple pulses (in situ: n_p_ = 8, in vivo: n_p_ = 15) to avoid overlap with the spillover effect.

We initially observed an increase in the LATEST effect with a variation of the high-frequency field strength. However, with further growth, the effect decreased due to the spillover effect [53]. Further, our simulation studies showed that at 3 T, the LATEST effect of about 0.45% as measured by MTR_asym_ in in situ and in vivo muscles could be expected. In contrast, results of 4–7% can be detected at 7 T [16]. 

Following our in silico and in vitro experiments, in which we successfully demonstrated a concentration dependence of LATEST, our optimized pre-saturation modules were as follows: n_p_ = 8, t_p_ = 50 ms, and B_1_ = 0.6 µT (in situ, T = 20 °C); and n_p_ = 15, t_p_ = 50 ms, and B_1_ = 0.5 µT (in vivo, T = 37 °C). Thus, the acquisition time of the optimized in vivo lactate-CEST sequence was 5:07 min (in situ: 3:07 min), followed by another 3:41 min for WASSR. Hence, our optimized in vivo CEST imaging protocol with a sequence duration of 8:48 min is of interest for research and clinical applications. In addition, we optimized the LATEST imaging protocol for 3 T MRI scanners, the field strength commonly used in clinical practice. However, compared to high-field MRI systems with 7 T and more, the optimized imaging protocol must be considered less sensitive; in particular, higher field strengths allow for volumetric multi-slice approaches due to the higher SNR, whereas our measurements record only one slice [54]. Furthermore, the in vitro experiments showed that pH changes in a physiological range of 7.0–7.3 can be neglected compared to the change in lactate levels. Our results are consistent with those of DeBrosse et al. In both studies, the LATEST effect is maximized at a pH value of 7.0 [16].

Using our optimized LATEST protocol, we observed a significant correlation of the LATEST effect in situ as a function of injected lactate concentration. Because this study was the first of its kind to investigate LATEST imaging at 3 T, we chose a wide range of lactate concentrations, from 0 to 40 mM, to evaluate the feasibility of this technique. However, in the clinical context, changes in the range of 0–12 mM are of primary interest [40].

We measured, at rest, MTR_asym_ values of 0.09 ± 0.02% in the calves of healthy volunteers. Considering our in vitro and in situ studies, this corresponds to a concentration below the detection limit of ~5–10 mM. This is consistent with reported concentrations of 3.8 ± 1.1 mM of endogenous lactate in muscle under resting conditions [55]. Consequently, contributions of other metabolites in the lactate-specific frequency range seem to be small in vivo. After exertional exercise, we observed a significant increase of MTR_asym_ values in the frequency ranges of 0.5–1 ppm and 1.5–2 ppm. This indicated an increase in lactate and creatine concentrations in response to exercise. The observed effect steadily decreased to resting levels over the following 22 min. Considering our in vitro and in situ experiments, lactate levels of about 20 mM were present in the muscles after “all-out” exertional exercises. This observation is in line with results of Goodwin et al., who measured lactate values of 15–25 mM in the blood serum about 3–8 min after exercise [56]. However, it should be kept in mind that in addition to changes in fractional lactate concentration, changes in pH also influence the observed LATEST effect. Street et al. demonstrated a change in pH from 7.38 (resting) to 7.04 (post-exercise) [41]. In the in vitro experiments, we observed a slight increase in the LATEST effect from 0.56% to 0.61% between pH values of 7.3 and 7.0 at a temperature of 37 °C. In comparison, the LATEST effect increased from 0.03% (0 mM lactate) to 0.56% (40 mM lactate). Thus, MTR_asym_ levels measured immediately after exercise could be slightly elevated due to the pH change, but this does not explain the substantial changes. The change in lactate concentration should therefore be considered a substantial factor.

However, even though our study demonstrates the feasibility of LATEST imaging at 3 T, some limitations have to be considered. First, OH-protons of lactate have a resonance frequency close to water, so low B_1_ field strengths and short pulse durations are necessary to ensure no overlap with the larger effect of direct water saturation. Second, this method requires high B_0_ and B_1_ field homogeneity, which was achieved using a special 15-channel transmit/receive coil with 15 integrated preamplifiers, positioned centrally in the MR scanner’s bore. Third, our statistical power was limited due to the small sample size. Thus, we only observed significant differences between concentrations below the detection limit, i.e., 0 and 5 mM, and concentrations above the detection limit, i.e., 20 and 40 mM. Nevertheless, we observed significant correlations of the MTR_asym_ values with the injected lactate concentrations, so it is reasonable to assume that further significant differences between lower lactate concentrations would be seen with larger sample sizes. Fourth, the transferability of our in situ study results to the in vivo configuration is limited. In subsequent studies, the lower detection limit of about 5–10 mM as observed in vitro and in situ should be verified. Fifth, no further measurements of lactate concentration were performed in situ and in vivo to validate changes in lactate concentration. Furthermore, the assumed in situ concentrations are based on a homogeneous distribution of lactate within the study volume; the exact distribution of lactate due to different fiber architectures has not been validated in separate studies. Therefore, the assumed in situ concentrations must be considered approximate values, which, however, show good agreement with the in vitro experiments. In a subsequent study ΔMTR_asym_ values could be correlated with blood lactate concentrations to investigate the sensitivity of lactate CEST imaging. Sixth, we considered only the MTR_asym_ method in this study; however, this method does not allow for the isolated detection of individual CEST effects and is affected by an overlap of different pools and attenuation of the detected CEST effect to nuclear Overhauser enhancement (NOE) effects. Techniques such as multi-pool Lorentz analysis or multi-pool Bloch–McConnell fitting can be used to detect different CEST effects separately and in isolation from each other. However, these methods require a sufficiently broad and accurately sampled Z-spectrum, which leads to a substantial increase in acquisition time, and therefore would not be suitable for our experiments designed to demonstrate the feasibility of LATEST imaging in the presence of longitudinal changes of lactate concentration. Seventh, we did not perform reproducibility measurements and test-retest experiments due to our standardization of study regions.

## 5. Conclusions

This study demonstrates that LATEST imaging at 3 T is feasible. Based on numerical Bloch–McConnell simulations, we optimized saturation pulse sequences for in situ and in vivo LATEST imaging. Subsequently, we successfully validated our simulation results by systematically varying lactate concentrations and temperatures in situ and in vitro. Finally, we were able to detect exertional exercise-induced lactate-weighted signal in vivo using our optimized CEST sequence, which may allow for the quantification of altered lactate levels using non-invasive MRI across a broad spectrum of diseases in the future.

## Figures and Tables

**Table 1:** Magnetic resonance imaging (MRI) acquisition parameters.

	Coronal	Sagittal	T1	T2	LATEST- CEST	LATEST- CEST	WASSR ^d^
	T2w TSE	T1w TSE	Mapping ^a^	Mapping	In Situ ^b^	In Vivo ^c^	
**Orientation**	cor	sag	cor	cor	cor	cor	cor
**TE (ms)**	78	11	10	^e^	5.76	5.76	5.76
**TR (ms)**	4720	650	6000	856	11	11	11
**Flip Angle (°)**	150	150	180	180	10	10	10
**Slices**	30	25	1	1	1	1	1
**Slice Thickness (mm)**	3	3	6	6	6	6	6
**FoV (mm × mm)**	160 × 160	320 × 320	160 × 160	160 × 160	160 × 160	160 × 160	160 × 160
**Image matrix (pixels)**	512 × 512	704 × 704	128 × 128	128 × 128	128 × 128	128 × 128	128 × 128
**Pixel Size (mm × mm)**	0.3 × 0.3	0.5 × 0.5	1.3 × 1.3	1.3 × 1.3	1.3 × 1.3	1.3 × 1.3	1.3 × 1.3
**Duration (min:s)**	2:12	1:54	8:38	1:02	3:37	5:07	3:41

Abbreviations: TE—echo time; TR—repetition times; TI—inversion time; FoV—field of view; T2w—T2 weighted; T1w—T1 weighted; SE—spin echo; CEST—chemical exchange saturation transfer; WASSR—water saturation shift referencing; B_1_—high-frequency field strength. ^a^—TI = 25, 50, 100, 500, 1000, 2000, and 3000 ms. ^b^—B_1_ = 0.6 µT, t_p_ = 50 ms; t_d_ = 50 ms, and n_p_ = 8. ^c^—B_1_ = 0.5 µT, t_p_ = 50 ms; t_d_ = 50 ms, and n_p_ = 15. ^d^—B_1_ = 0.2 µT, t_p_ = 25 ms; t_d_ = 0 ms; n_p_ = 1. ^e^—9.7, 19.4, 29.1, 38.8, 48.5, 58.2, 67.9, 77.6, 87.3, 97, 116.4, 126.1, 135.8, 145.5, 155.2, and 164.9 ms.

**Table 2:** Parameter and boundary settings for the optimization of the pre-saturation module.

	In Situ Measurements	In Vivo Measurements
**Pool parameters:**		
Exchange rate k_sw_	350 Hz	550 Hz
Δω [C_3_H_4_O_3_^−^]	0.6 ppm	0.4 ppm
c [H_2_O]	88 M	88 M
c [C_3_H_4_O_3_^−^]	15 mM	15 mM
T_1_ [H_2_O]\T_2_ [H_2_O]	856 ms\69 ms	1412 ms\50 ms
T_2_ [C_3_H_4_O_3_^−^]	240 ms	240 ms
**Boundaries:**		
B_1_	[0; 1.5] µT	
tp	[0; 0.6] ms	
n_p_	[1; 20]	

Abbreviations: c [X]—concentration of metabolite X; B_1_—high-frequency field strength; t_p_—pulse duration; t_d_—inter-pulse delay; n_p_—number of pulses.

**Table 3:** Absolute changes of the asymmetry magnetization transfer ratio (ΔMTR_asym_) values under various lactate concentrations in situ. Absolute changes were determined by relating the post-injection ΔMTR_asym_ values to the pre-injection ΔMTR_asym_ values. The non-parametric Friedman test was used to determine whether the different lactate concentrations differed significantly concerning ΔMTR_asym_.

Concentrations (mM)	ΔMTR_asym_ (%)			
	0–0.5 ppm	0.5–1 ppm	1–1.5 ppm	1.5–2 ppm
**0**	0.09 ± 0.94	−0.09 ± 0.17	0.03 ± 0.08	0.07 ± 0.19
**5**	0.09 ± 0.91	−0.05 ± 0.10	0.2 ± 0.45	0.09 ± 0.25
**20**	0.04 ± 0.72	0.41 ± 0.17	0.02 ± 0.05	−0.05 ± 0.06
**40**	−0.26 ± 0.19	0.70 ± 0.32	0.19 ± 0.25	−0.08 ± 0.09
*p*-value	0.228	**<0.001**	0.137	0.017

## Data Availability

Data and evaluation scripts can be provided by the authors upon reasonable request.

## Supplementary Materials

The following supporting information can be downloaded at: https://www.mdpi.com/article/10.3390/tomography8030106/s1, Figure S1: Illustration of the custom-made MRI-compatible phantom used for the in vitro study; Figure S2: Representative Z-spectra and MTR_asym_ curves for the three studied scenarios in vitro, in situ, and in vivo; Figure S3: Longitudinal changes of MTR_asym_ values of the human calves before and immediately after exertional exercise in the frequency ranges 0–0.5 ppm and 1–1.5 ppm.
