# Supplementary material for: Chemical Exchange Saturation Transfer for Lactate-Weighted Imaging at 3 T MRI: Comprehensive In Silico, In Vitro, In Situ, and In Vivo Evaluations
Source: Tomography. 2022 May 7;8(3):1277–92. doi: 10.3390/tomography8030106 (PMC9149919; doi:10.3390/tomography8030106)
Supplement: Supplementary file 1 [file tomography-08-00106-s001.zip › tomography-1673615-supplementary.pdf]

Chemical exchange saturation transfer for lactate-weighted imaging at 3 T MRI: Comprehensive *in-silico*, *in-vitro*, *in-situ*, and *in-vivo* evaluations

Karl Ludger Radke, Daniel B. Abrar, Miriam Frenken, Lena Marie Wilms, Benedikt Kamp, Matthias Boschheidgen, Patrick Liebig, Alexandra Ljimani, Timm Joachim Filler, Gerald Antoch, Sven Nebelung, Hans-Jörg Wittsack, Anja Müller-Lutz

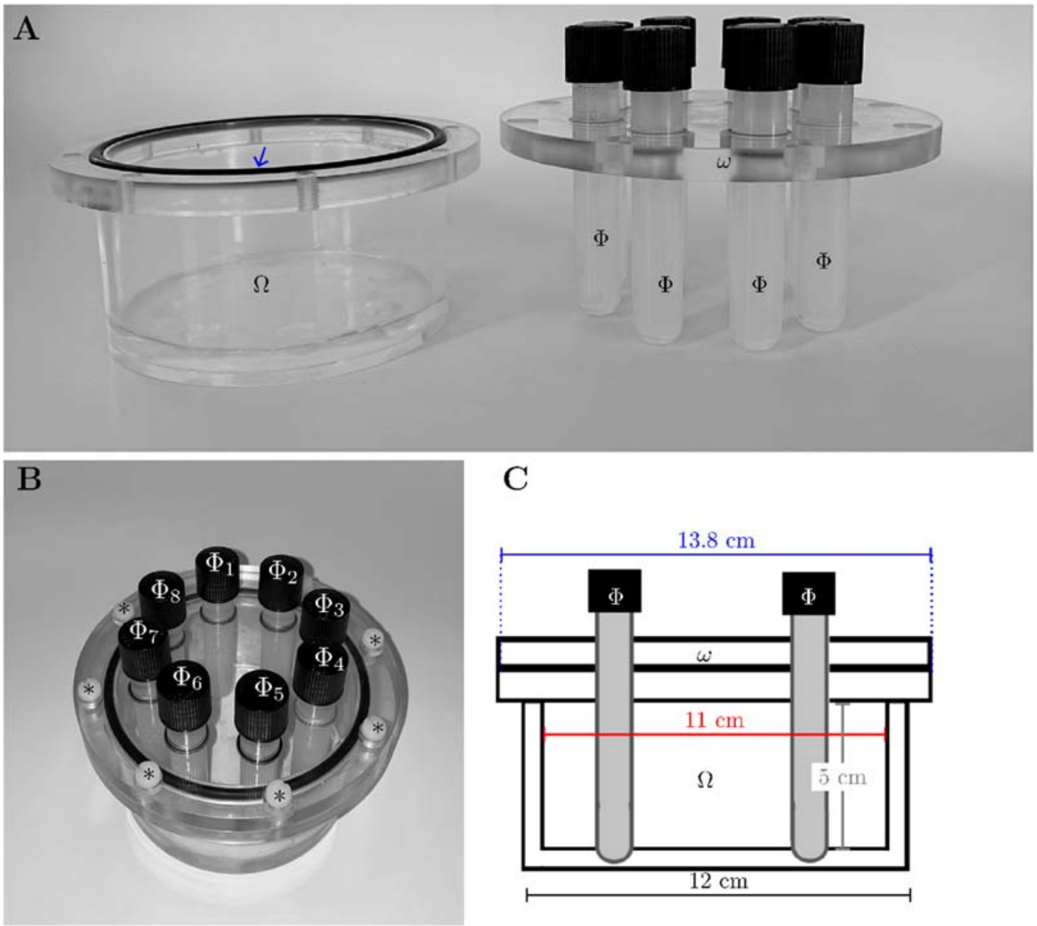

Supplementary Figure S1. Illustration of the custom-made MRI-compatible phantom used for the in vitro study. (A) The phantom consisted of two parts, a lower water reservoir (Ω) and a lid (ω) into which 8 test tubes (Φ) could be inserted. A sealing ring (blue arrow) was placed between the two parts to prevent water from escaping during the measurement. (B) Using 8 MRI-compatible screws (*), the two parts were additionally fixed to each other. The eight test tubes (Φ_1 to Φ_8) were filled clockwise, from Φ_1 to Φ_8 with 1.5% agarose and increasing lactate concentration (e.g., 0, 1, 5, 10, 15, 20, 30 and 40 mM). The lid of the test tubes could be unscrewed to measure the temperature in the phantom. (C) Schematic side view of the phantom with the exact dimensions.

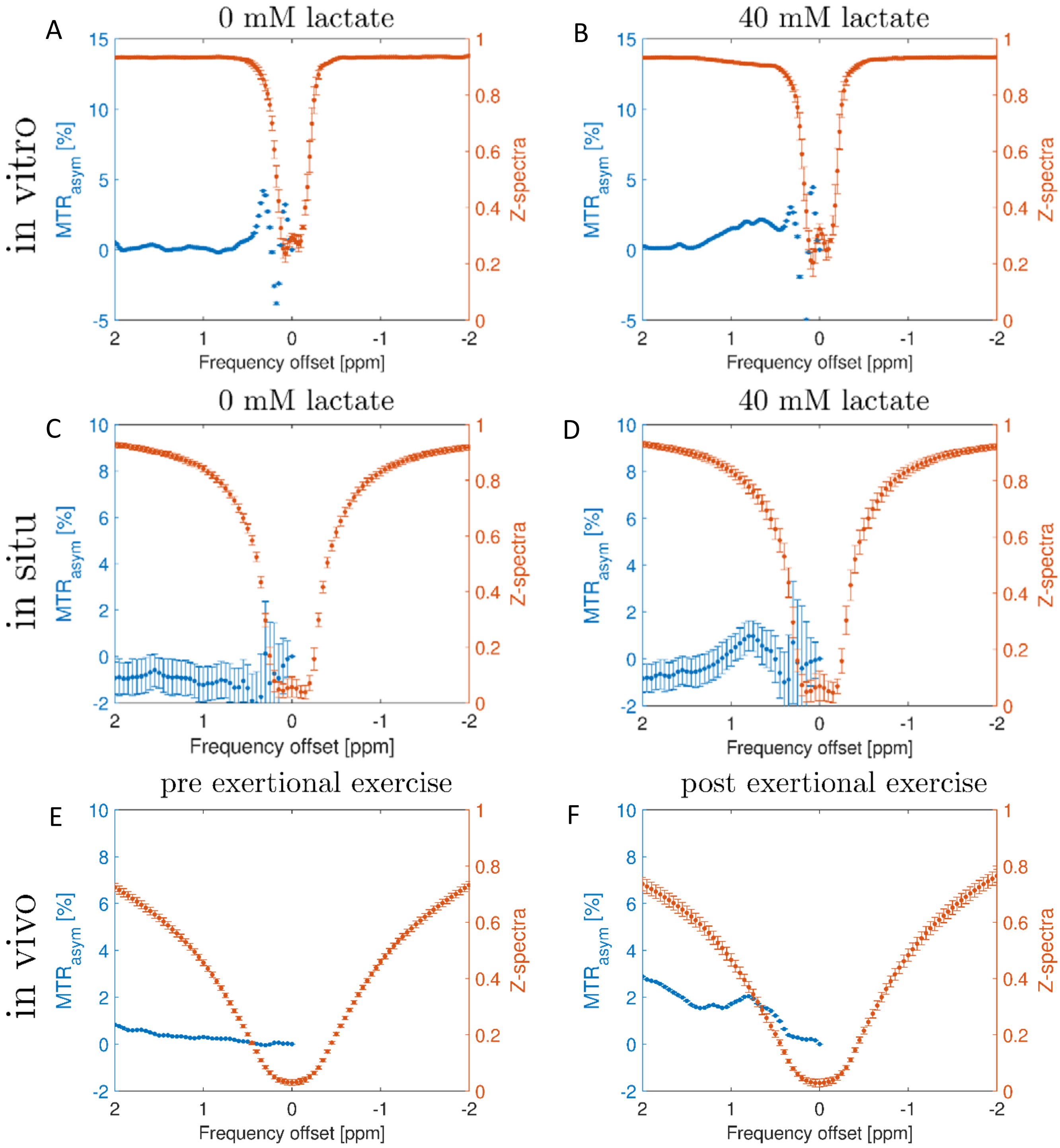

Supplementary Figure S2. Visualization of representative Z-spectra and the resulting MTR$_{asym}$ curves under systematic variation of lactate concentration in vitro (A, B), in situ (C, D), and in vivo (E, F).

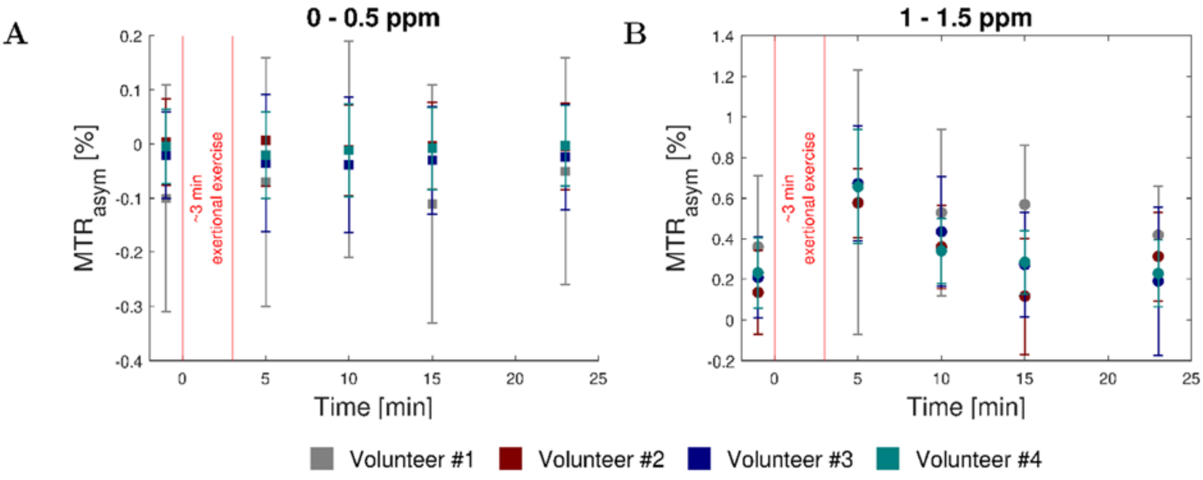

Supplementary Figure S3. Longitudinal changes of MTR_{asym} values (given as mean and standard deviation) of the four human calves (different colors) before and immediately after exertional exercise. (A) In the range, 0-0.5 ppm, no temporal changes of MTR_{asym} values are observed. (B) In the range, 1-1.5 ppm, a slight increase of MTR_{asym} values after exertional exercise can be observed.
